# Real‐Life Impact of Enfortumab Vedotin or Chemotherapy in the Sequential Treatment of Advanced Urothelial Carcinoma: The ARON‐2 Retrospective Experience

**DOI:** 10.1002/cam4.70479

**Published:** 2025-02-20

**Authors:** Mimma Rizzo, Franco Morelli, Yüksel Ürün, Sebastiano Buti, Se Hoon Park, Maria T. Bourlon, Enrique Grande, Francesco Massari, Johannes Landmesser, Alexandr Poprach, Hideki Takeshita, Giandomenico Roviello, Zin W. Myint, Lazar Popovic, Andrey Soares, Halima Abahssain, Patrizia Giannatempo, Javier Molina‐Cerrillo, Lorena Incorvaia, Samer Salah, Annalisa Zeppellini, Fernando Sabino Marques Monteiro, Camillo Porta, Shilpa Gupta, Matteo Santoni

**Affiliations:** ^1^ Medical Oncology Unit Azienda Ospedaliera Universitaria Consorziale Policlinico di Bari Bari Italy; ^2^ Medical Oncology Unit IRCCS Casa Sollievo della Sofferenza Foggia Italy; ^3^ Department of Medical Oncology, Faculty of Medicine Ankara University Ankara Turkey; ^4^ Department of Medicine and Surgery University of Parma Parma Italy; ^5^ Medical Oncology Unit University Hospital of Parma Parma Italy; ^6^ Samsung Medical Center Sungkyunkwan University School of Medicine Seoul Korea; ^7^ Department of Hemato‐Oncology, Instituto Nacional de Ciencias Medicas y Nutricion Salvador Zubiran Universidad Panamericana Mexico City Mexico; ^8^ Department of Medical Oncology MD Anderson Cancer Center Madrid Madrid Spain; ^9^ Department of Medical and Surgical Sciences (DIMEC) University of Bologna Bologna Italy; ^10^ Medical Oncology IRCCS Azienda Ospedaliero‐Universitaria di Bologna Bologna Italy; ^11^ Klinik für Urologie Universitätsklinikum Schleswig‐Holstein Campus Lübeck Lübeck Germany; ^12^ Department of Comprehensive Cancer Care Masaryk Memorial Cancer Institute Brno Czech Republic; ^13^ Faculty of Medicine Masaryk University Brno Czech Republic; ^14^ Department of Urology, Saitama Medical Center Saitama Medical University Saitama Japan; ^15^ Department of Health Sciences, Section of Clinical Pharmacology and Oncology University of Florence Florence Italy; ^16^ Division of Medical Oncology, Department of Internal Medicine, Markey Cancer Center University of Kentucky Lexington Kentucky USA; ^17^ Faculty of Medicine, Oncology Institute of Vojvodina University of Novi Sad Novi Sad Serbia; ^18^ Hospital Israelita Albert Einstein São Paulo Brazil; ^19^ Latin American Cooperative Oncology Group–LACOG Porto Alegre Brazil; ^20^ Medicine and Pharmacy Faculty, National Institute of Oncology, Medical Oncology Unit Mohamed V University Rabat Morocco; ^21^ Medical Oncology Department Fondazione IRCCS Istituto Nazionale dei Tumori Milan Italy; ^22^ Department of Medical Oncology Hospital Ramón y Cajal Madrid Spain; ^23^ Department of Precision Medicine in Medical, Surgical and Critical Care (Me.Pre.C.C.), Section of Medical Oncology University of Palermo Palermo Italy; ^24^ Department of Adult Medical Oncology King Fahad Specialist Hospital‐Dammam Dammam Saudi Arabia; ^25^ Niguarda Cancer Center Grande Ospedale Metropolitano Niguarda Milan Italy; ^26^ Hospital Sírio‐Libanês Brasília Brazil; ^27^ Chair of Oncology, Interdisciplinary Department of Medicine University of Bari “Aldo Moro” Bari Italy; ^28^ Taussig Cancer Institute Cleveland Clinic Cleveland Ohio USA; ^29^ Medical Oncology Unit Macerata Hospital Macerata Italy

**Keywords:** ARON‐2 study, chemotherapy, enfortumab vedotin, NCT05290038, pembrolizumab, real‐world data, sequencing, urothelial carcinoma

## Abstract

**Background:**

Recently, a plethora of novel systemic agents have been incorporated into the therapeutic armamentarium of advanced urothelial carcinoma (aUC). The antibody–drug conjugate (ADC), enfortumab vedotin (EV), has demonstrated relevant clinical benefit in patients with aUC refractory to platinum and immune‐checkpoint inhibitor (ICI) therapy. Our study provides a retrospective, international, real‐world analysis comparing the effectiveness of EV to chemotherapy in this setting.

**Methods:**

The data were extracted from the medical records of patients treated with EV or chemotherapy following pembrolizumab for recurrent or progressive aUC after platinum‐based chemotherapy. Patients were assessed for overall survival (OS), progression‐free survival (PFS), overall response rate (ORR) and duration of response (DoR).

**Results:**

Our analysis included 247 patients treated with EV (88, 36%) or chemotherapy (159, 64%). Median OS was 9.1 months (95%CI 7.2–10.7) in the overall study population, 13.6 months (95%CI 10.0–31.0) in patients receiving EV and 6.8 months (95%CI 6.0–8.9) in patients receiving chemotherapy (*p* < 0.001). The OS benefit of EV was not affected by primary tumour site and histology, metastatic sites, type of first platinum‐based chemotherapy or response to pembrolizumab. In the EV cohort, the median PFS was significantly longer (8.8 months [95%CI 6.5–17.0] vs. 3.0 months [95%CI 2.6–3.7]) and the ORR was significantly higher (56% vs. 23%) than in the chemotherapy cohort.

**Conclusions:**

The results of our international analysis of real‐world data confirm the effectiveness of EV in the sequential strategy of aUC patients who have received prior platinum‐based chemotherapy and anti‐PD‐1 pembrolizumab, regardless of commonly considered prognostic factors.

**Trial Registration:** ClinicalTrials.gov identifier: NCT05290038

## Graphical abstract

The findings of our international retrospective analysis, designated the ARON‐2 study, substantiate the pivotal role of the antibody–drug conjugate enfortumab vedotin (EV) in the therapeutic strategy for previously treated advanced urothelial carcinoma (aUC). EV has been demonstrated to be more efficacious than chemotherapy in terms of overall survival (OS), progression‐free survival (PFS) and overall response rate (ORR) in patients with aUC who received prior platinum‐based therapy and immunotherapy, regardless of their clinical and disease characteristics.

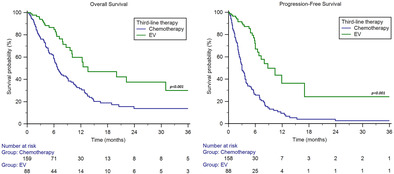

## 1 Introduction

Advanced Urothelial Carcinoma (aUC) is an aggressive disease with one of the lowest 5‐year survival rates among the most common cancers [1]: ranging from a minimum of 6.8% for the worst prognosis patients with visceral metastases to a maximum of 21% for the best prognosis patients with only lymph node metastases [2]. Over time, in real world case series, drop‐out rates after first‐line systemic treatment have remained consistent: only 17%–37% of aUC patients undergoing first‐line treatment receive a second‐line therapy and unfortunately no more than 6%–12% receive a third‐line therapy [3, 4, 5, 6, 7]. The main reason for UC patients' discontinuation is the limited benefit/tolerability profile of subsequent treatment options [8].

Based on the latest clinical trial results, the treatments options currently available for patients progressing after platinum‐based chemotherapy and PD‐1/PD‐L1 checkpoint inhibitors (ICIs) are: enfortumab vedotin (EV) [9, 10], sacituzumab govitecan (SG) (only in the US) [11] and erdafitinib (only for patients with FGFR2/3 alterations) [12].

The EV‐301 study (NCT03474107) [9, 10], a global, multicentre, open‐label, randomised, phase III trial, compared EV with standard chemotherapy (paclitaxel, docetaxel or vinflunine) in 608 patients previously treated with platinum‐based chemotherapy and a PD‐1/PD‐L1 inhibitor. The median OS was 12.88 and 8.97 months in the EV and chemotherapy arms, respectively, with 51.5% and 39% of patients surviving 12 months in the EV and chemotherapy arms, respectively [9]. Furthermore, the median PFS was longer in the EV arm (5.55 months) than in the chemotherapy arm (3.71 months) and the ORR was higher in the EV arm (40.6% vs. 17.9%).

Chemotherapy (paclitaxel, docetaxel, vinflunine, or platinum‐based) is a further treatment option, although clinical benefit is usually limited. More recently, a large retrospective multicentre real‐world study showed efficacy of platinum‐based chemotherapy re‐challenge after ICI in locally advanced or metastatic UC [13]. ORR (29.2%, 95% CI: 21.9 to 36.6) and PFS (4.9 months, 95% CI: 4.1 to 5.5) after ICI are more promising than those historically reported for second‐line chemotherapy and associated with an acceptable safety profile [13].

Furthermore, enrolment in randomised clinical trials should always be considered for patients who have progressed on chemotherapy and immunotherapy.

The ARON‐2 study was a multicentre, international, retrospective study designed to collect global real‐world data on the effectiveness of pembrolizumab in patients with relapse or progression after platinum‐based chemotherapy.

In this paper, we evaluated sequential treatment strategies following pembrolizumab and their outcomes in different countries included in the real‐world, international ARON‐2 study.

## 2 Patients and Methods

### 2.1 Study Population

The ARON‐2 study population included patients aged ≥ 18 years with a cytological and/or histological confirmed diagnosis of recurrent or progressing aUC after platinum‐based therapy and treated with pembrolizumab between 1 January 2016 to 1 April 2024 at 47 institutes in 17 countries worldwide (Table S1).

All patients included in the sequential treatment strategy analysis had known data on: age, gender, Eastern Cooperative Oncology Group‐Performance Status (ECOG‐PS), primary tumour location (upper vs. lower tract), tumour histology, time of surgery and radiotherapy on primary tumour and/or metastases, setting (neoadjuvant vs. adjuvant vs. metastatic setting) and type of first platinum‐based chemotherapy (cisplatin vs. carboplatin‐based), magnitude and duration of response to first‐line chemotherapy, onset of metastatic disease (synchronous vs. metachronous disease), metastasis sites, magnitude and duration of response to immunotherapy, date of last follow‐up or death.

Clinical data were retrospectively and locally extracted, at each participating centre, from the patients' medical records. The pathological information were abstracted from pathology reports for clinical use. Radiologists or investigators at each institution evaluated response to pembrolizumab according to Response Evaluation Criteria In Solid Tumours version 1.1 (RECIST 1.1 criteria) [14].

Patients with missing information and patients who received ICI in combination, targeted therapy or investigational drugs in the therapeutic sequence were excluded from our analysis.

### 2.2 Study Endpoints

Disease response to third systemic treatment was determined in each centre, referring to the Response Evaluation Criteria in Solid Tumours version 1.1 (RECIST 1.1) as complete response (CR), partial response (PR), stable disease (SD) and progressive disease (PD). Overall Response Rate (ORR) was calculated by the sum of CR and PR.

Progression‐free survival (PFS) was calculated from the first administration of third‐line systemic treatment to documented disease progression or death from any cause, whichever occurred first. Patients without disease progression or death or lost at follow‐up at the time of the analysis were censored at the last follow‐up visit.

Overall survival (OS) was calculated from the start of third systemic treatment to death from any cause. Patients alive or lost at follow‐up at the time of the analysis were censored at the last follow‐up visit. Duration of response (DoR) was calculated from the time of the first imaging assessing the achievement of CR or PR with third‐line EV or chemotherapy to documented disease progression or death from any cause, whichever occurred first.

### 2.3 Statistical Analysis

The Kaplan–Meier method with Rothman's 95% confidence intervals (CI) was used to estimate survival curves of OS and PFS. Comparisons between survival curves were performed using the log‐rank test. Cox proportional hazards models were adopted to compare the multivariable effects on patients' survival and to calculate hazard ratios (HRs) and 95% confidence intervals (CIs). Data available from the ARON dataset on sex, age, ECOG‐PS, smoking attitude, histology, surgery, time to metastatic disease, sites of metastases and type of third‐line therapy were included in the multivariable analysis.

To assess the potential differences between variables in Table 1 and between ORR and 1y‐OS and 1y‐PFS rates, Fisher's exact test was performed to assess statistically significant associations between dual categorical variables, chi‐square test for multiple categorical variables. The level of significance was set to 0.05, and all *p* values were two‐sided.

**TABLE 1:** Baseline patient characteristics after chemotherapy (CT) and pembrolizumab (P) according to treatment group (chemotherapy rechallenge vs. enfortumab vedotin).

Characteristics	Total 247 (%)	Chemotherapy 159 (%)	Enfortumab vedotin 88 (%)	*p*
Median age (years)	68	69	67	—
≥ 70 year	119 (48)	78 (49)	41 (47)	0.779
Sex				
Male	183 (74)	121 (76)	62 (70)	0.426
Female	64 (26)	38 (24)	26 (30)	
ECOG performance status (after CT and P)				
0–1	193 (78)	121 (76)	72 (82)	0.386
2–3	54 (22)	38 (24)	16 (18)	
Tumour histology				
Urothelial carcinoma	208	136 (86)	72 (82)	0.442
Squamous cell carcinoma	18	9 (6)	9 (10)	0.298
Other histological variant	21	14 (8)	7 (8)	1.000
Site of primary tumour				
Upper urinary tract	63 (26)	37 (23)	26 (30)	0.263
Lower urinary tract	184 (74)	122 (77)	62 (70)	
Presenting with metastatic disease at diagnosis	79 (32)	58 (36)	21 (24)	0.065
Prior surgery on primary tumour	156 (63)	95 (60)	61 (69)	0.185
Metastatic sites (after CT and P)				
Distant lymph nodes	160 (65)	112 (70)	48 (55)	0.029
Lung	82 (33)	55 (35)	27 (31)	0.549
Liver	50 (20)	38 (24)	12 (14)	0.072
Bone	56 (23)	35 (22)	21 (24)	0.738
Brain	2 (1)	2 (1)	0 (0)	1.000
First‐line chemotherapy: type of platinum				0.007
Cisplatin	158 (64)	91 (57)	67 (75)	
Carboplatin	89 (36)	68 (43)	21 (25)	
Pembrolizumab: setting				
Patients relapsed within < 1 year since neoadjuvant/adjuvant CT	133 (54)	61 (38)	72 (82)	< 0.001
Patients progressed during I‐line platinum‐based CT	114 (46)	98 (62)	16 (18)	
Best response to pembrolizumab				
Complete response (CR)	7 (3)	3 (2)	4 (5)	0.282
Partial response (PR)	47 (19)	30 (19)	17 (19)	1.000
Stable disease (SD)	59 (24)	39 (25)	20 (23)	0.744
Progressive disease (PD)	134 (54)	87 (54)	47 (53)	0.888

*Note: p*‐values were calculated by Fisher's exact test.

MedCalc version 19.6.4 (MedCalc Software, Broekstraat 52, 9030 Mariakerke, Belgium) was used for the statistical analyses.

## 3 Results

### 3.1 Patients Population

In the ARON‐2 dataset we included 1039 patients treated with pembrolizumab for recurrent or progressing aUC after platinum‐based therapy. From them we selected 247 patients (23.8%) treated with chemotherapy (159, 64%) or EV (88, 36%) after progression to platinum‐based chemotherapy and pembrolizumab. The selection process is summarised in Figure S1. The median follow‐up from the start of third‐line EV or chemotherapy was 15.8 months (95%CI 8.7–44.6).

Baseline patient characteristics are listed in Table 1. The median age at the start of third systemic treatment was 68 years (range 41–86). The study population consisted of 74% male and 26% female patients. In total, 78% of patients (193 patients) who were eligible for third‐line systemic treatment exhibited favourable general clinical conditions, defined according to the Eastern Cooperative Oncology Group (ECOG) Performance Status (PS) as 0 or 1. The majority of patients (74%) had urothelial carcinoma of the bladder, while 26% had upper urinary tract carcinoma. At the time of diagnosis, 32% of patients had metastatic disease. According to metastatic sites patients were distributed as follows: 65% with lymph node metastases only, 33% with lung metastases, 20% with liver metastases, 23% with bone metastases and 1% with brain metastases.

The chemotherapy regimen following progression to pembrolizumab are summarised in Table 2. Twenty‐one percent of patients received an additional platinum‐based chemotherapy treatment (2% platinum monotherapy, 19% platinum‐containing polychemotherapy) and 76% received another chemotherapeutic agent in monotherapy (31% paclitaxel, 30% vinflunine, 9% docetaxel, 6% gemcitabine).

**TABLE 2:** Type of chemotherapy following pembrolizumab therapy.

Regimen	Chemotherapy 159 (%)
Monotherapy	
Cisplatin	2 (1)
Carboplatin	2 (1)
Paclitaxel	49 (31)
Docetaxel	15 (9)
Vinflunine	48 (30)
Gemcitabine	9 (6)
Polichemotherapy	
Carboplatin and gemcitabine	19 (12)
Carboplatin and paclitaxel	11 (7)
MVAC	4 (3)

Forty‐seven patients (19%) received further therapies (27 patients in the chemotherapy group and 20 in the EV group), which consisted in chemotherapy in 45 patients (two patients were enrolled into clinical trials).

### 3.2 Survival Outcomes

**FIGURE 1:**
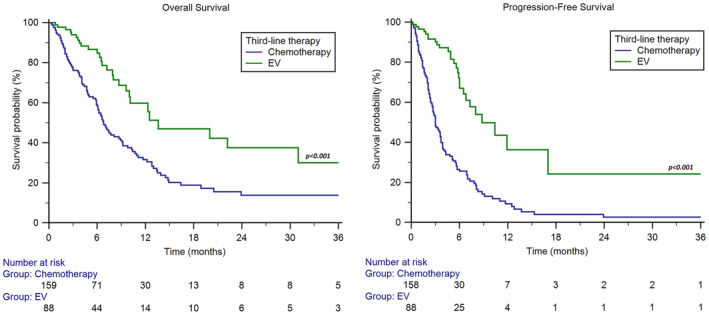
Overall survival and progression‐free survival of patients treated with chemotherapy or enfortumab vedotin following pembrolizumab for advanced UC.

Overall, median OS was 9.1 months (95%CI 7.2–10.7). Median OS was 6.8 months (95%CI 6.0–8.9) in patients receiving chemotherapy and 13.6 months (95%CI 10.0–31.0) for those treated with EV (*p* < 0.001, Figure 1), with a 6‐months‐OS rate of 58% vs. 85% (*p* < 0.001) and a 1y‐OS rate of 32% vs. 60% (*p* < 0.001).

**FIGURE 2:**
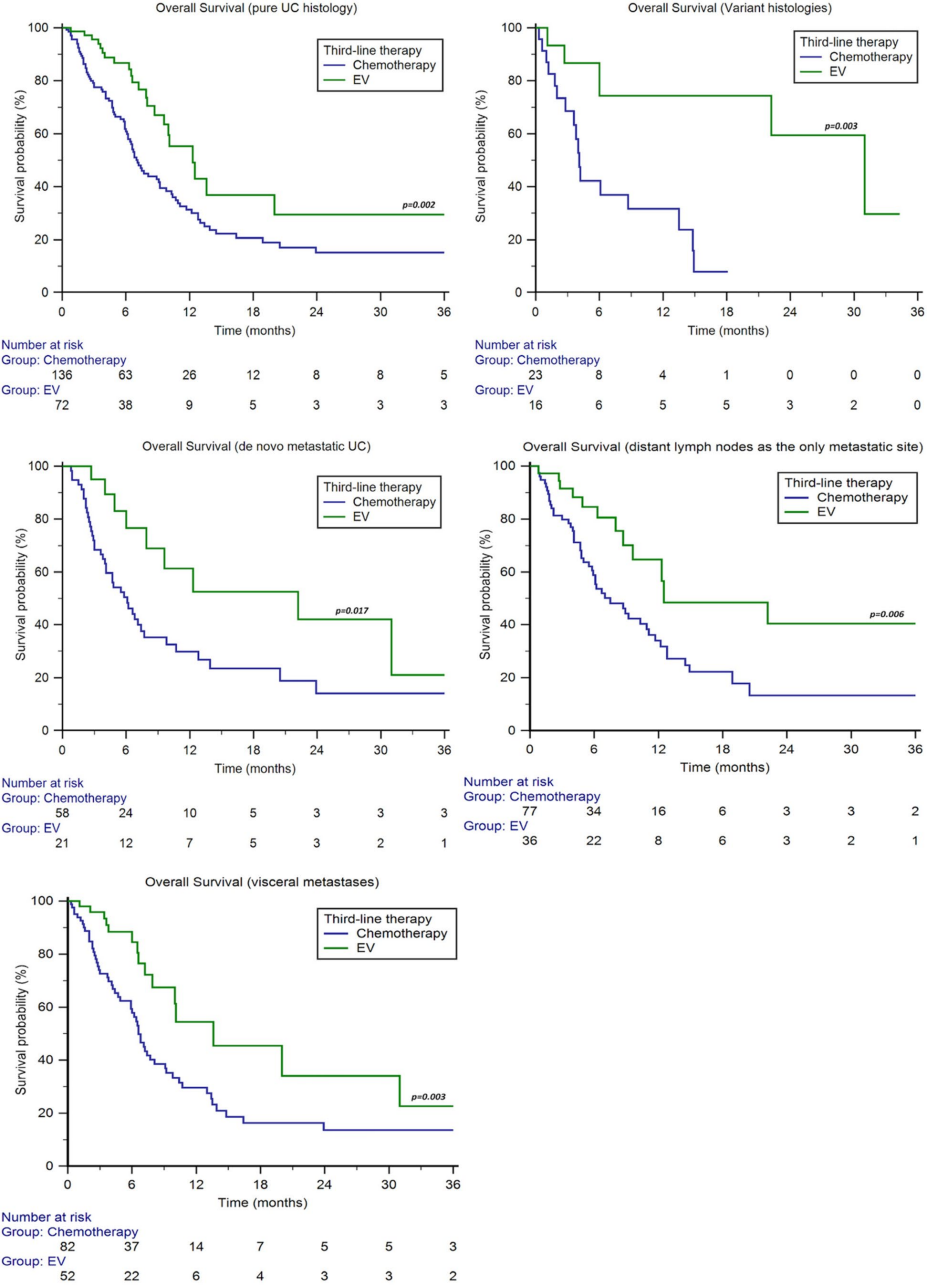
Overall survival in patients stratified by tumour histology, metastatic status, presence of distant lymph node‐only metastases and visceral metastases.

Patients with pure UC histology showed longer median OS with EV (12.3 months, 95%CI 9.6–20.0 vs. 7.1 months, 95%CI 6.2–9.2, *p* = 0.002, Figure 2), as well as patients with variant histologies (31.0 months, 95%CI 6.0–31.0 vs. 4.1 months, 95%CI 2.8–13.5, *p* = 0.003, Figure 2).

Patients with de novo metastatic disease showed longer median OS with EV (22.2 months, 95%CI 6.0–31.0 vs. 6.1 months, 95%CI 4.0–7.7, *p* = 0.017, Figure 2).

We stratified patients into two subgroups: only metastases to distant lymph nodes and visceral metastases. The median OS was longer with EV in both subgroups (patients with only lymph node metastases: 12.5 months, 95%CI 8.7–22.2 vs. 7.5 months, 95%CI 5.5–11.1, *p* = 0.006; patients with visceral metastases: 13.6 months, 95%CI 7.9–31.0 vs. 6.6 months, 95%CI 5.9–9.1, *p* = 0.003, Figure 2).

No significant differences were found in patients with lung (chemotherapy: 6.6 months, 95%CI 5.9–9.2 vs. EV: 13.6 months, 95%CI 7.9–31.0, *p* = 0.077), liver (chemotherapy: 6.1 months, 95%CI 4.0–10.7 vs. EV: 13.6 months, 95%CI 3.8–13.6, *p* = 0.157) or bone metastases (chemotherapy: 6.1 months, 95%CI 3.7–9.8 vs. EV: 9.6 months, 95%CI 3.8–22.2, *p* = 0.236).

**FIGURE 3:**
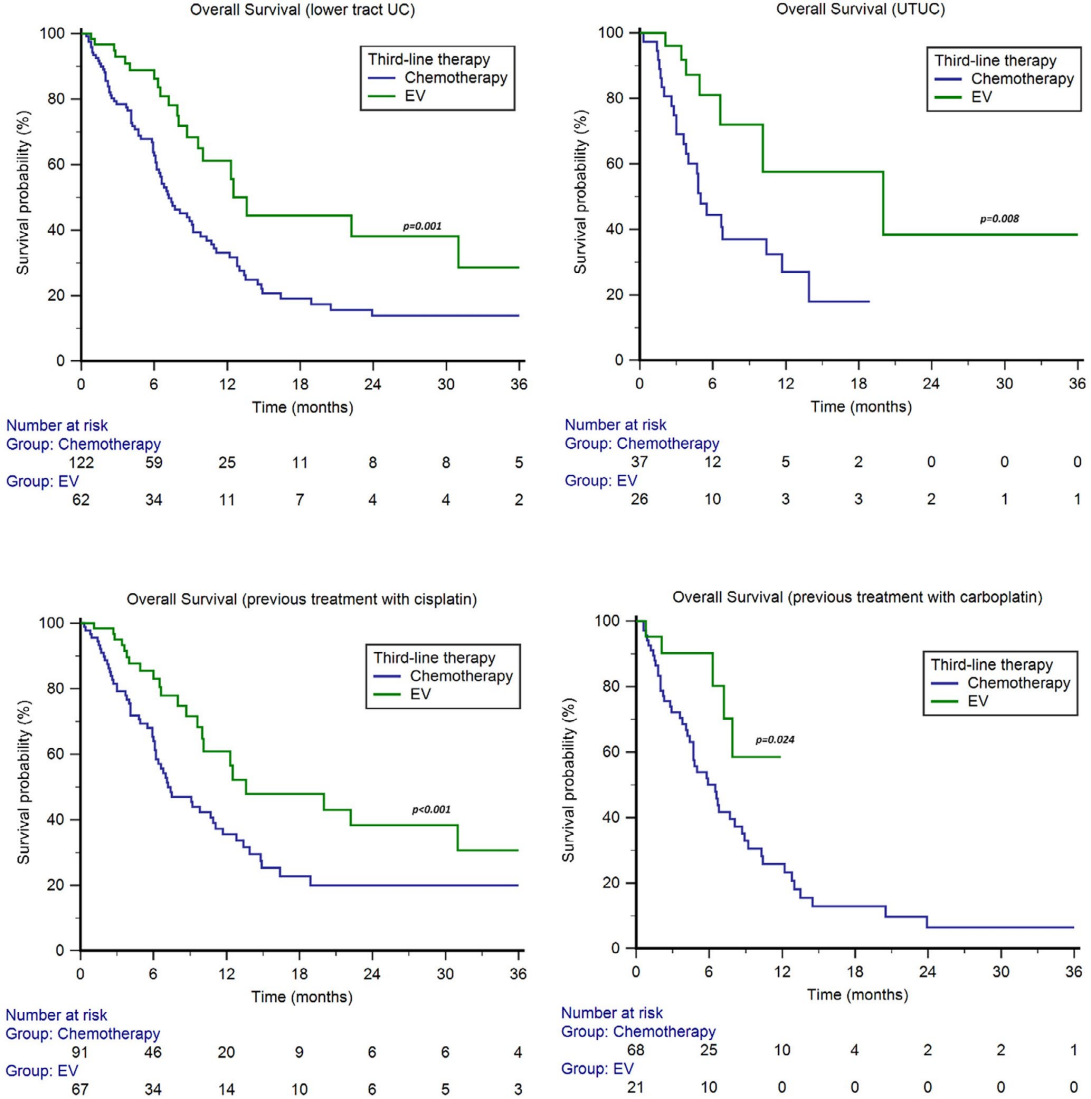
Overall survival of patients treated with chemotherapy or enfortumab vedotin (EV) following pembrolizumab, stratified by site of primary tumour and type of platinum‐based first‐line therapy or adjuvant/neoadjuvant therapy.

The median OS with EV was higher in both patients with lower tract UC (13.6 months, 95%CI 9.6–31.0, vs. 7.2 months, 95%CI 6.2–9.2, *p* = 0.001, Figure 3) and UTUC (20.0 months, 95%CI 6.6–20.0, vs. 5.0 months, 95%CI 3.6–10.4, *p* = 0.008, Figure 3).

In patients treated with cisplatin‐based first‐line therapy or adjuvant/neoadjuvant chemotherapy, the median OS was 13.6 months (95%CI 10.1–31.0) for EV and 7.4 months (95%CI 6.2–11.1) for chemotherapy (*p* < 0.001, Figure 3), with a 1y‐OS rate of 61% vs. 36%, respectively (*p* < 0.001). In patients treated with carboplatin‐based first‐line therapy or adjuvant/neoadjuvant chemotherapy, the median OS was NR (95%CI NR–NR) in the EV subgroup and 6.5 months (95%CI 4.4–8.7) in the chemotherapy subgroup, with a 1y‐OS rate of 59% vs. 26%(*p* < 0.001, Figure 3).

**FIGURE 4:**
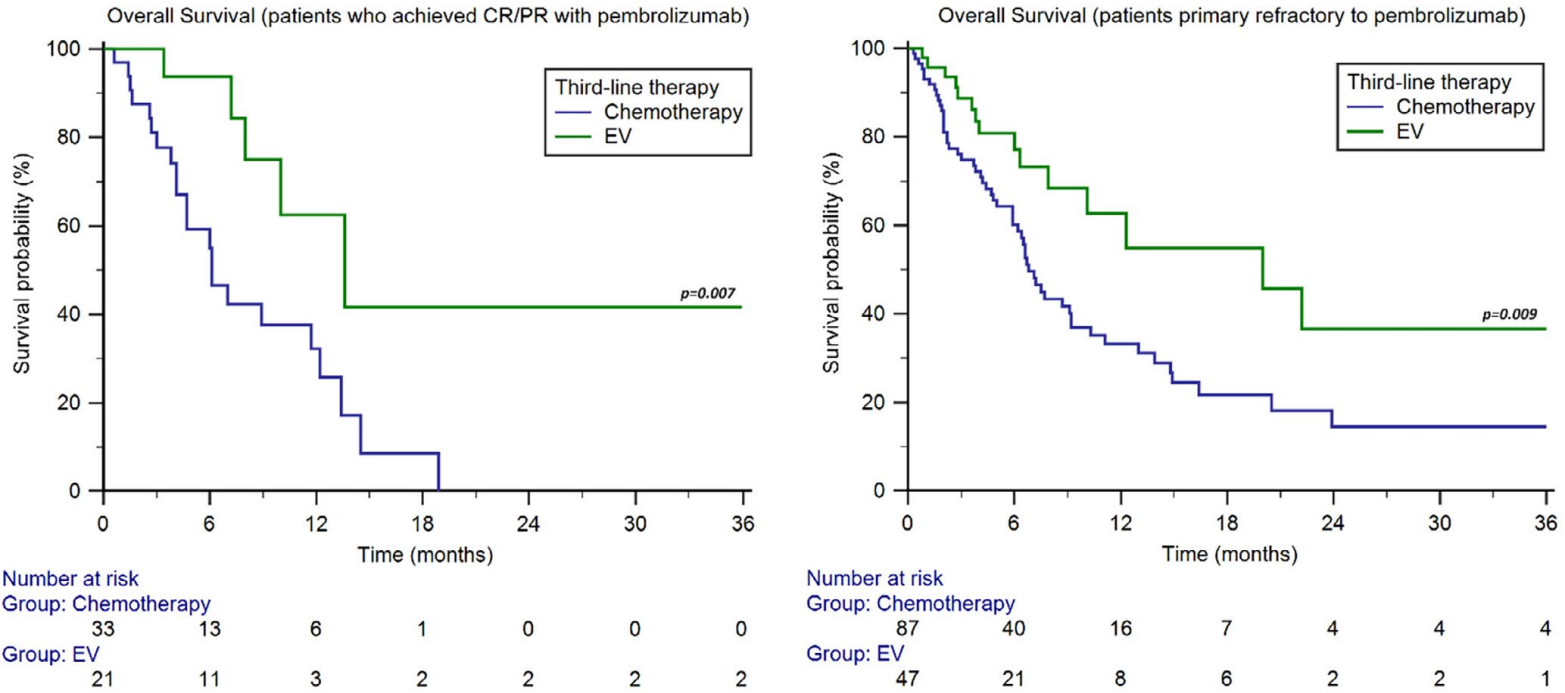
Overall survival of patients treated with chemotherapy or enfortumab vedotin following pembrolizumab, stratified by the response to immunotherapy.

We also performed stratification of the study population based on the response to pembrolizumab. In the 54 patients who achieved CR/PR with pembrolizumab, the median OS was 6.1 months (95%CI 4.1–18.9) with chemotherapy and 13.6 months (95%CI 7.2–13.6) with EV (*p* = 0.007, Figure 4). On the other hand, in the 134 patients who were primary refractory to immunotherapy, showing PD as best response to pembrolizumab, the median OS was 6.8 months (95%CI 5.9–9.2) with chemotherapy and 20.0 months (95%CI 7.9–22.2) with EV (*p* = 0.009, Figure 4).

The median PFS was 4.9 months (95%CI 3.7–5.8). The median PFS was 3.0 months (95%CI 2.6–3.7) in patients receiving chemotherapy and 8.8 months (95%CI 6.5–17.0) for those treated with EV (*p* < 0.001, Figure 1), with a 6‐months‐PFS rate of 26% vs. 67% (*p* < 0.001) and a 1y‐PFS rate of 9% vs. 36% (*p* < 0.001).

### 3.3 Response Rate

In the third‐line setting of the overall study population, we observed 5% CR, 30% PR, 31% SD and 34% PD. In patients receiving chemotherapy following pembrolizumab, we reported 1% of CR, 22% of PR, 34% of SD and 43% of PD, with an ORR of 23% (95_%CI 17–30) (Table 3). In patients treated with EV, we observed 12% of CR, 44% of PR, 27% of SD and 17% of PD, with an ORR of 56% (95_%CI 46–66, *p* < 0.001, Table 3).

**TABLE 3:** Response rate according to treatment group (chemotherapy vs. enfortumab vedotin).

	Chemotherapy (*N* = 159) (%)	Enfortumab vedotin (*N* = 89) (%)
Best response according to RECIST 1.1		
Complete response (CR)	1	12
Partial response (PR)	22	44
Stable disease (SD)	34	27
Progressive disease (PD)	43	17
Objective response rate (CR + PR)	23	56

In carboplatin‐pretreated patients, we observed 4% of CR, 28% of PR, 25% of SD and 43% of PD. In this subgroup, patients receiving chemotherapy showed 0% of CR, 21% of PR, 27% of SD and 52% of PD, while patients treated with EV reported 16% of CR, 47% of PR, 21% of SD and 16% of PD. The ORR was higher in patients receiving EV (63%, 95%CI 42–84 vs. 21%, 95%CI 11–31, *p* < 0.001).

In patients who achieved CR or PR with third‐line therapy, the median DoR was 8.0 months (95%CI 4.2–11.0) for chemotherapy and 17.0 months (95%CI 11.9–17.0) for EV (*p* < 0.001).

### 3.4 Prognostic Factors

At univariate and multivariable analyses, ECOG‐PS ≥ 2 and the choice of third‐line therapy were significant predictors of OS (Table 4).

**TABLE 4:** Univariable and multivariable analyses of overall survival in patients treated with enfortumab vedotin versus chemotherapy following platinum‐based chemotherapy and pembrolizumab.

Overall survival (ECOG‐PS 2)	Univariable cox regression		Multivariable cox regression	
	HR (95%CI)	*p*	HR (95%CI)	*p*
Sex (females vs. males)	1.28 (0.88–1.88)	0.200		
Age ≥ 70 year (yes vs. no)	0.91 (0.64–1.29)	0.595		
ECOG‐PS ≥ 2 vs. 0–1	3.25 (2.09–5.06)	**< 0.001**	2.63 (1.67–4.15)	**< 0.001**
Smokers vs. no‐smokers	0.85 (0.58–1.24)	0.401		
Histology (mixed vs. pure UC)	1.05 (0.77–1.45)	0.743		
Upper vs. lower urinary tract	1.09 (0.73–1.64)	0.669		
Synchronous metastatic disease (yes vs. no)	1.20 (0.84–1.71)	0.306		
Distant lymph node (yes vs. no)	0.88 (0.60–1.31)	0.531		
Lung metastases (yes vs. no)	0.99 (0.69–1.44)	0.969		
Liver metastases (yes vs. no)	1.20 (0.79–1.82)	0.384		
Bone metastases (yes vs. no)	1.27 (0.85–1.90)	0.237		
Brain metastases (yes vs. no)	1.76 (0.42–7.17)	0.431		
Third‐line therapy (EV vs. chemotherapy)	0.43 (0.28–0.66)	**< 0.001**	0.50 (0.33–0.78)	**0.002**

*Note:* Bold values indicate *p* < 0.05.

## 4 Discussion

In our multicentre, international, retrospective ARON‐2 study [15, 16, 17, 18], 23.8% (247 patients) of the 1039 aUC patients included were treated with third‐line treatment after platinum‐based chemotherapy and pembrolizumab. A recent retrospective cohort study analysing data from 7260 aUC patients included in the US Flatiron Health database [7], reported a significantly lower percentage of patients receiving third‐line treatment (11.8%) compared to our study. The above US findings are consistent with other cohort studies conducted in other countries [3, 4, 5, 6], but differ from our worldwide study findings. Several factors may have contributed to the lower drop‐out rate in our real‐world case series, including: (I) selection and time‐lead bias: only patients with favourable clinical conditions and early access to pembrolizumab after platinum‐based chemotherapy were included in our retrospective study; (II) moderate socioeconomic and treatment disparities in our overall international population; (III) optimised selection and management of first‐ and second‐line treatments in the participating countries; (IV) a higher proportion of US patients receiving more innovative and effective first‐line treatments, due to earlier access to novel treatments compared to other countries around the world.

Recent case series suggest an increased efficacy of salvage chemotherapy after the integration of pembrolizumab into aUC patients' treatment strategy in comparison to salvage chemotherapy results in the pre‐immunotherapy era: ORR of salvage chemotherapy after pembrolizumab ranges from 17% to 66.7% and PFS from 2.5 to 7.9 months [13, 19, 20, 21]. Results from these retrospective case series and the standard chemotherapy control arm of the EV‐301 trial [9, 10] were better than historical data on second‐line chemotherapy with taxanes [22, 23] or vinflunine [24].

In the 159‐patient cohort from our study receiving chemotherapy after pembrolizumab, the results were consistent with the literature data: ORR was 23% and median PFS was 3.0 months (95%CI 2.6–3.7). Although the clinical implications of these findings are limited, they confirm that indirect immune modulation by cytotoxic agents [25, 26] could enhance CD8+ T‐cell activity induced by PD‐1/PD‐L1 inhibitors [27] and promote anti‐tumour responses.

In this regard, ADCs as tissue‐specific chemotherapeutics represent a sophisticated strategy to maximise immunogenic cell death [28] and improve the recruitment and activity of CD8+ effector T cells in the tumour core [29]. From June 2021 [30], the drug conjugated antibody, EV, represents a therapeutic option of unquestionable effectiveness, capable of significantly extending the life expectancy of patients previously treated with platinum‐based chemotherapy and a PD‐1/PD‐L1 inhibitor. In the EV‐301 study, the aforementioned clinically significant benefits in terms of OS and PFS were observed in multiple subgroups [9], and were confirmed at a median follow‐up of approximately 2 years [10].

Our results were comparable to those of the EV‐301 trial results. Median OS was 13.6 months for patients receiving EV and 6.8 months for those treated with chemotherapy (Figure 1), with a 1‐year OS rate of 60% versus 32%, respectively. Median PFS was 8.8 months in the EV cohort and 3.0 months in the chemotherapy cohort (Figure 1), with a 1‐year PFS rate of 9% versus 36%, respectively. In addition, the ORR in the EV group was significantly higher (56% vs. 23%) and the DoR was significantly longer (17 vs. 8 months) than in the chemotherapy group. The outcomes we observed with EV are more favourable than those reported in other recent case series comparing EV and re‐challenging chemotherapy after platinum agents and pembrolizumab in real‐world clinical practice [31, 32]. The availability of EV may have encouraged clinicians to consider third‐line treatment earlier than in the past, when conventional treatment options (chemotherapeutic agents) were not supported by solid evidence of efficacy and burdened by a considerable toxicity profile. Indeed, 82% of patients who received EV in the third‐line systemic treatment setting exhibited a favourable overall clinical condition, defined as a Performance Status (PS) of 0 or 1 according to the Eastern Cooperative Oncology Group (ECOG) criteria.

In the retrospective real‐world UNITE study, 566 patients from 17 US sites were treated with EV monotherapy and exhibited an ORR of 49%, a median PFS of 5.8 months, and a median OS of 12.2 months at the 21‐month median follow‐up from the start of EV treatment [33, 34].

In our case series, the OS advantage of EV was irrespective of tumour histology, primary tumour site, time of metastatic disease, metastasis sites and disease burden (Figures 2 and 3). Multivariable analysis has confirmed that the survival rate of patients treated with EV in the third line is significantly longer than chemotherapy, regardless of the platinum agent used in the first line (cisplatin vs. carboplatin) (Figure 3) and the response to second‐line immunotherapy (pembrolizumab) (Figure 4). This suggests that there is no cross‐resistance between any of these agents and EV.

Although previous retrospective studies have indicated inferior outcomes of EV in variant histologies than in pure UC [35, 36], our findings suggest that EV may be a more effective treatment than chemotherapy in this specific population.

Among patients who received adjuvant/neoadjuvant or first‐line carboplatin‐based chemotherapy in our study [37], median OS was not reached in the EV subgroup and 6.5 months in the chemotherapy subgroup (Figure 3). The ORR was significantly higher in patients receiving EV (59% vs. 26%) and was consistent with the ORR (52%) achieved with EV in cisplatin‐ineligible patients enrolled in the multicentre, single‐arm, phase 2 EV‐201 trial [38, 39].

Treatment strategies for aUC currently vary widely within and between countries around the world, and more first‐line and post‐immunotherapy therapies will be broadly approved in the near future. Therefore, more data are needed to assess the effectiveness of different sequential treatment strategies for aUC and to identify potential prognostic factors to tailor treatment.

The main limitations of our work due to its retrospective nature are: (I) the EV cohort is relatively small in comparison to the standard chemotherapy cohort; (II) the first‐line treatment regimen is influenced by the inclusion period (prior to the approval of avelumab maintenance and immuno‐combinations in several countries); (III) limited information on patient and treatment characteristics is limited; (IV) toxicity data is unavailable; (V) survival bias of patients eligible for multiple lines of treatment; (VI) the lack of central radiological review can lead to misinterpretation of the response assessment; (VII) the lack of genomic evaluation of tumour tissue to ascertain molecular alterations such as FGFR mutation/fusion.

Although our results may not be fully representative of the global population, given the large sample size and the high number of centres and countries involved, they certainly offer a topic of reflection for healthcare professionals, patients and stakeholders.

## 5 Conclusions

The findings of our international real‐world analysis corroborate the effectiveness of enfortumab vedotin in sequential treatment strategies for patients with advanced urothelial carcinoma and provide further evidence that this antibody–drug conjugate leads to superior outcomes compared to chemotherapy.

## Author Contributions

**Mimma Rizzo:** conceptualization (lead), data curation (supporting), investigation (supporting), methodology (lead), project administration (supporting), supervision (lead), validation (lead), writing – original draft (lead), writing – review and editing (lead). **Franco Morelli:** writing – original draft (equal), writing – review and editing (equal). **Yüksel Ürün:** writing – review and editing (equal). **Sebastiano Buti:** writing – review and editing (equal). **Se Hoon Park:** writing – review and editing (equal). **Maria T. Bourlon:** writing – review and editing (equal). **Enrique Grande:** writing – review and editing (equal). **Francesco Massari:** writing – review and editing (equal). **Johannes Landmesser:** writing – review and editing (equal). **Alexandr Poprach:** writing – review and editing (equal). **Hideki Takeshita:** writing – review and editing (equal). **Giandomenico Roviello:** writing – review and editing (equal). **Zin W. Myint:** writing – review and editing (equal). **Lazar Popovic:** writing – review and editing (equal). **Andrey Soares:** writing – review and editing (equal). **Halima Abahssain:** writing – review and editing (equal). **Patrizia Giannatempo:** writing – review and editing (equal). **Javier Molina‐Cerrillo:** writing – review and editing. **Lorena Incorvaia:** writing – review and editing. **Samer Salah:** writing – review and editing. **Annalisa Zeppellini:** writing – review and editing. **Fernando Sabino Marques Monteiro:** writing – review and editing. **Camillo Porta:** writing – review and editing. **Shilpa Gupta:** writing – original draft (equal), writing – review and editing. **Matteo Santoni:** conceptualization (lead), data curation (lead), investigation (lead), methodology (lead), project administration (lead), supervision (lead), validation (lead), writing – original draft (lead), writing – review and editing (lead).

## Ethics Statement

The study protocol was approved on 28 September 2023, by the Ethical Committee of the coordinating centre (Marche Region, Italy, No. 2022 39/7875, Study Protocol ‘ARON 2 Study’) and by the Institutional Review Boards of participating centres.

## Consent

The informed consent with subsequent analysis of the follow‐up data was obtained from all participants. All authors have approved the manuscript for publication.

## Conflicts of Interest

Mimma Rizzo has received honoraria as a speaker/consultant by MSD, Merck Serono, Astrazeneca, Bristol Myers Squibb, Eisai and Gilead, all unrelated to the present paper. Francesco Massari has received research support and/or honoraria from Advanced Accelerator Applications, Astellas, Astra Zeneca, Bayer, BMS, Janssen, Ipsen, MSD, Pfizer, all unrelated to the present paper. Sebastiano Buti has received honoraria as speaker at scientific events and advisory role by BMS, Pfizer, MSD, Ipsen, Roche, Eli Lilly, AstraZeneca, Pierre‐Fabre, Novartis, Merck, Gentili, Astellas, all unrelated to the present paper. Enrique Grande has received honoraria for speaker engagements, advisory roles or funding of continuous medical education from Adacap, AMGEN, Angelini, Astellas, Astra Zeneca, Bayer, Blueprint, Bristol Myers Squibb, Caris Life Sciences, Celgene, Clovis‐Oncology, Eisai, Eusa Pharma, Genetracer, Guardant Health, HRA‐Pharma, IPSEN, ITM‐Radiopharma, Janssen, Lexicon, Lilly, Merck KGaA, MSD, Nanostring Technologies, Natera, Novartis, ONCODNA (Biosequence), Palex, Pharmamar, Pierre Fabre, Pfizer, Roche, Sanofi‐Genzyme, Servier, Taiho and Thermo Fisher Scientific, all unrelated to the present paper. Javier Molina‐Cerrillo has received research funding from Roche, Ipsen, Pfizer, and Janssen, travel support from Pfizer, Janssen, Ipsen and BMS and has been consultant or advisor to Ipsen, Roche, BMS, Pfizer, Sanofi, Janssen, Astellas, Eisai, Adium and MSD, all unrelated to the present paper. Fernando Sabino Marques Monteiro has received research support from Merck Sharp Dome and honoraria from Janssen, Ipsen, Bristol Myers Squibb and Merck Sharp Dome. Ownership: BIO, Brazilian Information Oncology, all unrelated to the present paper. Shilpa Gupta is a consultant for Bristol Myers Squibb, Merck, Pfizer, Gilead, Bayer, Seattle Genetics, is speaker for Bristol Myers Squibb and has Institutional research funding from Seatte Genetics, Pfizer, Merck, Bristol Myers Squibb, Roche, Novartis, Tyra Biosciences. Matteo Santoni has received research support and honoraria from Janssen, Bristol Myers Squibb, Ipsen, MSD, Astellas and Bayer, all unrelated to the present paper. Camillo Porta has received honoraria from Angelini Pharma, AstraZeneca, BMS, Eisai, Exelixis, Ipsen, Merck and MSD and has a protocol steering committee role for BMS, Eisai and MSD, all unrelated to the present paper.

## Supporting information


**Table S1.** List of countries participating to the ARON‐2 study.
**Figure S1.** Selection process from the ARON‐2 dataset.

## Data Availability

The datasets generated and/or analyzed during the current study are not publicly available due to patient data security but are available from the last author on reasonable request.
