# Supplementary material for: Real‐Life Impact of Enfortumab Vedotin or Chemotherapy in the Sequential Treatment of Advanced Urothelial Carcinoma: The ARON‐2 Retrospective Experience
Source: Cancer Med. 2025 Feb 20;14(4):e70479. doi: 10.1002/cam4.70479 (PMC11842279; doi:10.1002/cam4.70479)

**Supplementary Materials**

**Table S1.** List of Countries participating to the ARON-2 study.

| **List of Countries** | | |
| --- | --- | --- |
| Argentina | Austria | Belgium |
| Brazil | Czech Republic | Germany |
| Italy | Japan | Jordan |
| Korea | Maroc | Mexico |
| Poland | Serbia | Spain |
| Turkey | United States |  |

**Figure S1.** Selection Process from the ARON-2 dataset.


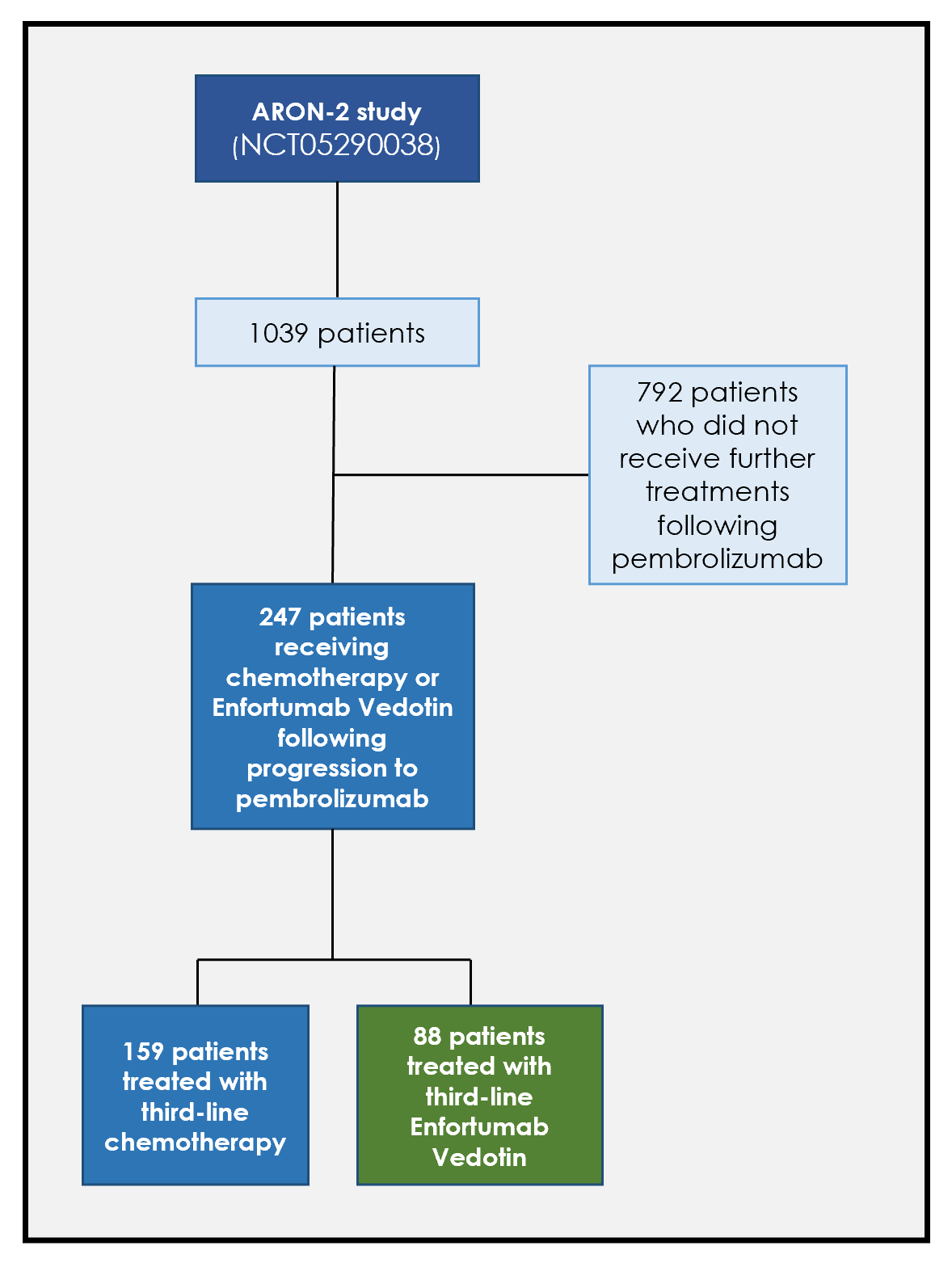

Supplement: Supplementary file 1 — Table S1. List of countries participating to the ARON‐2 study. Figure S1. Selection process from the ARON‐2 dataset. [file CAM4-14-e70479-s001.docx]
